# Sex-specific up-regulation of *p50-associated COX-2 extragenic RNA* (*PACER*) lncRNA in periodontitis

**DOI:** 10.1016/j.heliyon.2020.e03897

**Published:** 2020-05-13

**Authors:** Arezou Sayad, Soudeh Ghafouri-Fard, Bahareh Shams, Shahram Arsang-Jang, Leila Gholami, Mohammad Taheri

**Affiliations:** a Dental Research Center, Research Institute for Dental Sciences, Dental School, Shahid Beheshti University of Medical Sciences, Tehran, Iran; b Department of Medical Genetics, Shahid Beheshti University of Medical Sciences, Tehran, Iran; c Department of Periodontics, School of Dentistry, Shahid Beheshti University of Medical Sciences, Tehran, Iran; d Department of Biostatistics and Epidemiology, Cancer Gene Therapy Research Center, Faculty of Medicine, Zanjan University of Medical Sciences, Zanjan, Iran; e Department of Periodontics, School of Dentistry, Hamadan University of Medical Sciences, Hamadan, Iran; f Urogenital Stem Cell Research Center, Shahid Beheshti University of Medical Sciences, Tehran, Iran

**Keywords:** Cell biology, Molecular biology, Biocomputational method, Inflammation, Gene expression, Biomolecules, Genetic disorders, Immune disorder, THRIL, PACER, lncRNA, Periodontitis

## Abstract

A number of recent studies have shown dysregulation of some long non-coding RNAs (lncRNAs) in affected tissues or peripheral blood of patients with periodontitis. In the current study, we investigated the role of *TNF and HNRNPL related immunoregulatory* (*THRIL*) and *p50-associated COX-2 extragenic RNA* (*PACER*) lncRNAs in periodontitis. We assessed expression of these lncRNAs in 30 affected tissue, 30 control tissue samples, 23 blood samples from patients and 18 blood samples from healthy controls. Expression of *PACER* was higher in total blood samples of patients compared with controls (Posterior beta of RE = 5.143, P value = 0.001). However, when assessing its expression in a gender-based manner, the difference in the expression of this lncRNA was significant only among male subgroups (Posterior beta of RE = 7.16, P value < 0.0001). Moreover, expression of *PACER* was significantly higher in female subjects compared with male subjects (Posterior beta of RE = 3.098, P value < 0.0001). There was no significant difference in tissue expression of *PACER* between study subgroups. Expression of *THRIL* was not significantly different between blood/tissue samples of cases and controls. However, expression of this lncRNA was higher in blood of female subjects compared with male subjects (Posterior beta of RE = 4.353, P value = 0.002). Tissue expression of *THRIL* was correlated with blood levels of this lncRNA (r = 0.33, P < 0.0001) and with the tissue levels of *PACER* (r = 0.3, P < 0.0001). Moreover, blood levels of these lncRNAs were correlated with each other (r = 0.34, P < 0.0001). However, there was no significant correlation between blood and tissue levels of *PACER*. Expression of these lncRNAs were not correlated with age either in males or in females. Taken together, we demonstrated a sex-based up-regulation of *PACER* in blood samples of patients with periodontitis which implies possible participation of this lncRNA in the pathobiology of periodontitis.

## 1 Introduction

Periodontal disease is a chronic inflammatory condition that might lead to periodontal ligament loss and demolition of the neighboring alveolar bone. As the leading source of tooth loss, periodontitis is regarded as one of the two principal dangers to the oral health [1]. This disorder is more prevalent in men compared with women suggesting a potential involvement of gender in the pathogenesis of this condition [2]. Although bacteria are regarded as the main etiologic factor in the development of periodontitis, the pathogenesis of this disorder is principally inflammatory. However, the knowledge about the regulatory mechanisms of inflammation in periodontitis is not complete [3]. Some recent studies have elaborated the role of long non-coding RNAs (lncRNAs) in the pathobiology of periodontitis. Comprehensive assessment of lncRNA-associated competing endogenous RNA network has shown the role of *MALAT1*, *TUG1* and *FGD5-AS1* lncRNAs and cytokine-cytokine receptor, cell adhesion molecules and chemokine signaling pathway in the pathogenesis of periodontitis [4]. Moreover, abnormal initiation of the NF-κB pathway during inflammation has been shown to cause an imbalance in an lncRNA-miRNA regulatory network during the pathogenesis of periodontitis [5]. Besides, a microarray-based study of lncRNAs expression has shown differential expression of several of these transcripts in chronic periodontitis tissues compared with neighboring normal tissues, implying the roles of lncRNAs in the development of this disorder [6]. In the current study, we aimed at identification of the expression pattern of *TNF and HNRNPL related immunoregulatory* (*THRIL*) and *p50-associated COX-2 extragenic RNA* (*PACER*) lncRNAs in the periodontitis. *THRIL* has been firstly identified in an attempt to detect lncRNAs that are functionally linked with the activation of the innate immune response. This lncRNA was recognized as an inducer of TNF-α expression which exert its effect through binding with the heterogeneous nuclear ribonucleoprotein L (hnRNPL). Expression of this lncRNA has been correlated with the clinical course of the inflammatory condition of childhood, Kawasaki disease [7]. The lncRNA *PACER* has been shown to induce expression of cyclooxygenase (COX)-2 through hindering the functions of repressive NF-κB complexes [8]. Based on the role of TNF-α [9] and COX-2 [10] in the pathogenesis of periodontitis, *THRIL* and *PACER* are putative contributors in this inflammatory condition. Thus, we designed the current study to unravel their role in the pathogenesis of periodontitis.

## 2 Materials and methods

### 2.1 Blood and tissue samples

Gingival tissue specimens were acquired during periodontal flap surgeries from patients and crown lengthening or implant surgery from control subjects. Inclusion criteria for the periodontitis group were: the presence of at least 16 teeth, an age of 18 or older, chronic periodontitis (stages II to IV) with at least two remaining periodontal pockets in each sextant after nonsurgical periodontal treatment, probing depth of 5 mm or greater, bleeding on probing (BOP), and at least 3 mm of attachment loss needing surgical periodontal treatment [11]. Exclusion criteria were cigarette smoking, systemic disorders, history of antibiotic or anti-inflammatory drugs intake 3 months prior to surgery, pregnancy and breastfeeding. Patients were assessed by a periodontist to verify clinical and radiographic manifestations. Control samples were obtained persons who underwent crown lengthening or implant placement surgery. The sites were assessed by a periodontist and sites with negative BOP and less than 3 mm probing depths were included. Totally, 30 affected tissue, 30 control tissue samples, 23 blood samples from patients and 18 blood samples from healthy controls were assessed. Tissue and blood samples were obtained from the same individuals. Yet, we did not have blood sample of 7 patients and 12 control persons. None of the female subjects used oral contraceptives. The study protocol was approved by ethical committee of Shahid Beheshti University of Medical Sciences (Ethic Code: IR.SBMU.DRC.REC.1398.086). Patients signed informed consent forms.

### 2.2 Expression assays

Total RNA was isolated from all samples by using Hybrid-RTM blood RNA extraction kit (GeneAll, Seoul, South Korea). Next, cDNA was synthesized from extracted total RNA using the OneStep RT-PCR Series Kit (BioFact™, Seoul, South Korea). Relative expressions (RE) of *PACER* and *THRIL* were measured in all samples using the RealQ Plus 2x PCR Master Mix Green Without ROX™ PCR Master Mix (Ampliqon, Odense, Denmark). The StepOnePlus™ RealTime PCR equipment (Applied Biosystems, Foster city, CA, USA) was used for amplifications. *B2M* gene was used as normalizer. Table 1 summarizes the information about primers and PCR products.

**Table 1:** Summarized data of primers and PCR products.

lncRNA	Primer	Sequence	Product length
*THRIL*	Forward Reverse	aaggaggacacaacagat tagcagcaataagcaagc	100 bp
*PACER*	Forward Reverse	tggtcctaagcagttaccctgta accaaaataatccacgcatcagg	177 bp
*B2M*	Forward Reverse	agatgagtatgcctgccgtg gcggcatcttcaaacctcca	105 bp

### 2.3 Statistical methods

CT values and PCR efficiencies were retrieved from the real time PCR system software. Transcript levels of *PACER* and *THRIL* were compared between periodontitis patients and healthy subjects and between male subjects and female subjects using Bayesian regression model. RE of each gene was defined as Ln (PCR EfficiencyˆΔCt). The impacts of independent variables were adjusted. Analyses were executed in R 3.6.2 software with Rstan, ggplot 2 packages. Non-parametric quantile regression was executed using Bootstrap method and 100 iteration. Correlations between expressions of genes were appraised through calculation of Spearman correlation coefficients. P values less than 0.05 were considered as significant.

## 3 Results

### 3.1 Demographic data of study participants

Demographic data of periodontitis patients and healthy subjects are shown in Table 2.

**Table 2:** Demographic data of periodontitis patients and healthy subjects.

	Periodontitis patients	Healthy controls
Total Number of Tissue Samples	30	30
Female	19	14
Male	11	16
Age (Mean ± SD)	41.28 ± 2.7	38.8 ± 1.9
Total Number of Blood Samples	23	18
Female	15	11
Male	8	7
Age (Mean ± SD)	39.65 ± 3.1	37.91 ± 2.8

## 4 Results

**Figure 1:**
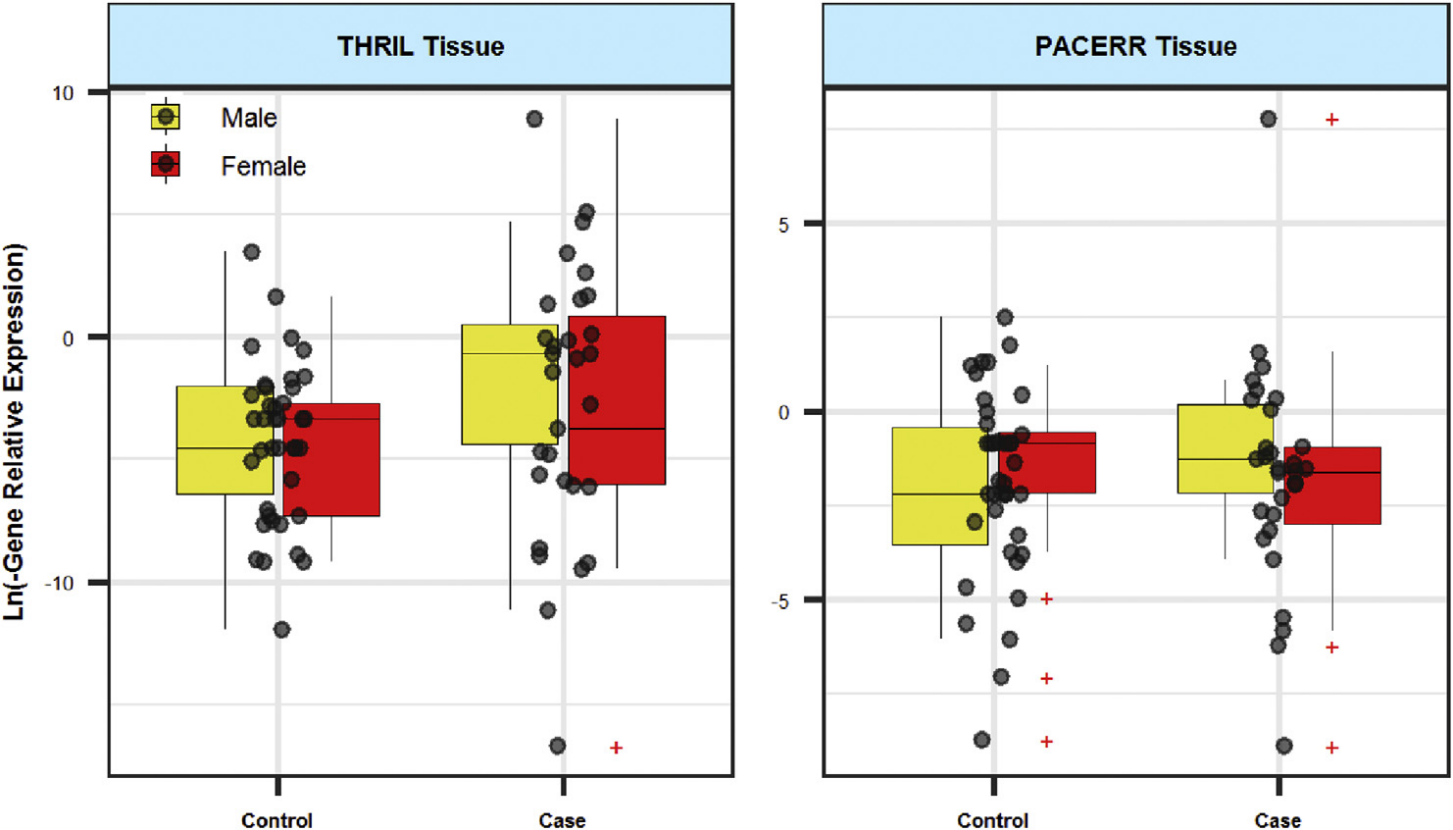
Relative expression of *THRIL* and *PACER* lncRNAs in gingival samples of cases and controls. Expression levels were measured using the Ln (PCR EfficiencyˆΔCt) formula. The interquartile range is shown. Outliers are shown by "+".

**Figure 2:**
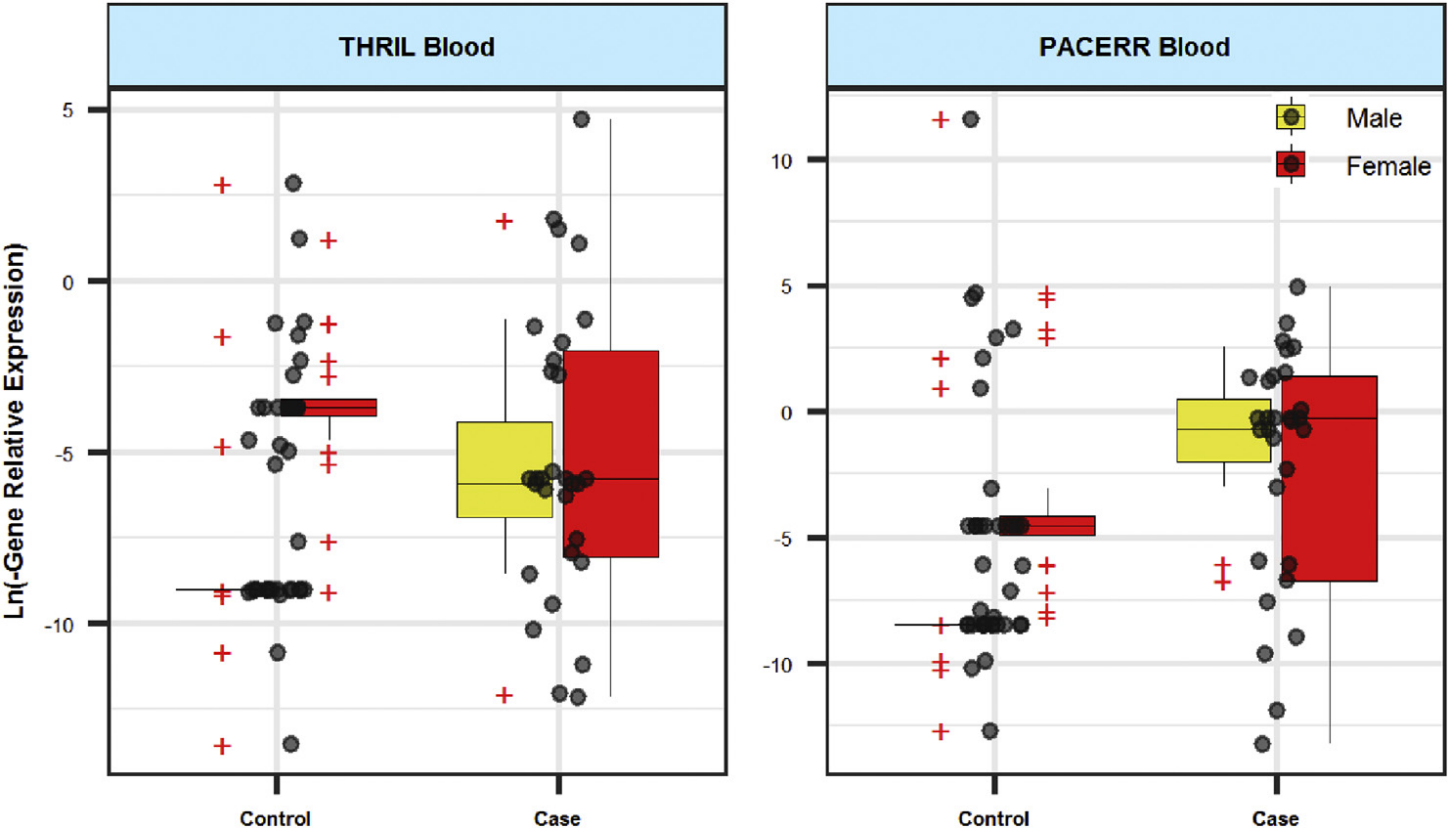
Relative expression of *THRIL* and *PACER* lncRNAs in blood samples of cases and controls. Expression levels were measured using the Ln (PCR EfficiencyˆΔCt) formula. The interquartile range is shown. Outliers are shown by "+".

Figures 1 and 2 show relative expression of *THRIL* and *PACER* lncRNAs in gingival and blood samples of patients with periodontitis and healthy subjects, respectively.

Expression of *THRIL* was not significantly different between blood/tissue samples of cases and controls. Moreover, when dividing study participants based on their gender, there was no significant difference between patients and sex-matched controls. However, expression of this lncRNA was higher in blood of female subjects compared with male subjects (Posterior beta of RE = 4.353, P value = 0.002). Table 3 shows the details of assessment of expression of this lncRNA in study subgroups. There were no sex differences in age. Based on our results, periodontitis was not age dependent.

**Table 3:** Relative expression of *THRIL* in tissues and blood samples of patients compared with controls (RE: relative expression, SE: standard error, CrI: credible interval).

Tissue						Blood			
Parameters and groups	Variable	Posterior Beta of RE	SE	P-Value	95% CrI for RE	Posterior Beta of RE	SE	P-Value	95% CrI for RE
Total	Case/Control	2.665	1.31	0.288	[-0.39, 4.94]	-0.101	1.32	0.375	[-2.41, 2.45]
	Gender (F/M)	0.123	0.9	0.452	[-1.58, 1.94]	4.353	0.72	0.002	[2.8, 5.52]
	Age (year)	0.003	0.06	0.999	[-0.09, 0.14]	0.002	0.03	0.999	[-0.05, 0.06]
Male	Case/Control	3.78	1.84	0.045	[0.1, 7.66]	2.484	0.89	0.001	[0.76, 4.19]
	Age (year)	0.101	0.1	0.917	[-0.09, 0.29]	0.019	0.03	0.999	[-0.03, 0.09]
Female	Case/Control	2.165	1.88	0.891	[-1.96, 5.27]	-1.663	1.27	0.189	[-3.92, 1.39]
	Age (year)	-0.053	0.08	0.999	[-0.21, 0.12]	-0.045	0.06	0.999	[-0.17, 0.04]

Expression of *PACER* was higher in total blood samples of patients compared with controls (Posterior beta of RE = 5.143, P value = 0.001). However, when assessing its expression in a gender-based manner, the difference in the expression of this lncRNA was significant only among male subgroups (Posterior beta of RE = 7.16, P value < 0.0001). Moreover, expression of *PACER* was significantly higher in female subjects compared with male subjects (Posterior beta of RE = 3.098, P value < 0.0001). There was no significant difference in tissue expression of *PACER* between study subgroups. Table 4 shows RE of *PACER* in tissues and blood samples of patients compared with controls.

**Table 4:** Relative expression of *PACER* in tissues and blood samples of patients compared with controls (RE: relative expression, SE: standard error, CrI: credible interval).

Tissue						Blood			
Parameters and groups	Variable	Posterior Beta of RE	SE	P-Value	95% CrI for RE	Posterior Beta of RE	SE	P-Value	95% CrI for RE
Total	Case/Control	-0.66	0.62	0.701	[-1.87, 0.56]	**5.143**	**0.98**	**0.001**	**[3.21, 7.07]**
	Gender (F/M)	0.528	0.56	0.278	[-0.69, 1.56]	**3.098**	**0.76**	**<0.0001**	**[1.53, 4.39]**
	Age (year)	0.049	0.03	0.488	[0, 0.104]	0.019	0.03	0.999	[-0.04, 0.09]
Male	Case/Control	-0.235	0.99	0.143	[-2.12, 1.66]	**7.16**	**1.29**	**<0.0001**	**[3.95, 9.35]**
	Age (year)	0.095	0.05	0.458	[0, 0.18]	0.002	0.04	0.999	[-0.07, 0.08]
Female	Case/Control	-0.741	0.72	0.132	[-2.22, 0.78]	3.361	1.69	<0.0001	[-1.05, 5.91]
	Age (year)	0.009	0.03	0.999	[-0.06, 0.08]	0.055	0.07	0.999	[-0.06, 0.23]

Bold denotes P-Value lesser than 0.05 as a significance.

**Figure 3:**
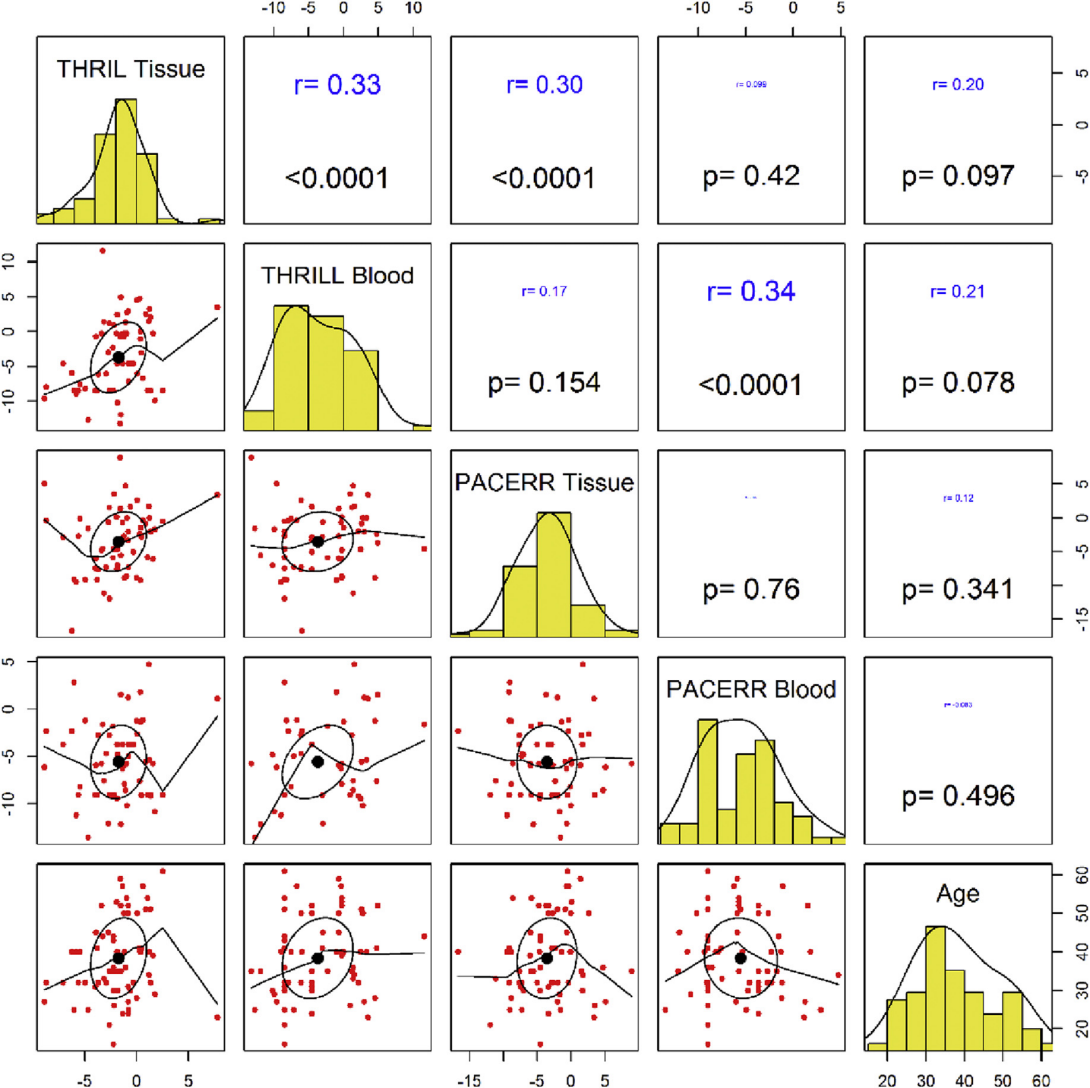
Correlation matrix plot showing correlation between blood/tissue expression levels of *THRIL* and *PACER* and age of study participants. The lower triangular matrix shows the bivariate scatter plots with a fitted smooth line. The upper triangular matrix demonstrates the Pearson correlation and P values.

Tissue expression of *THRIL* was correlated with blood levels of this lncRNA (r = 0.33, P < 0.0001) and with the tissue levels of *PACER* (r = 0.3, P < 0.0001). Moreover, blood levels of these lncRNAs were correlated with each other (r = 0.34, P < 0.0001). However, there was no significant correlation between blood and tissue levels of *PACER*. Expression of these lncRNAs were not correlated with age. Figure 3 shows the results of correlation analysis between blood/tissue expression levels of *THRIL* and *PACER* and age of study participants.

## 5 Discussion

The current investigation aimed at identification of the expression pattern of *THRIL* and *PACER* in blood and tissue samples of patients with periodontitis and healthy subjects. We detected higher expression of *PACER* in total blood samples of patients compared with controls. However, when assessing its expression in a gender-based manner, the difference in the expression of this lncRNA was significant only among male subgroups. *PACER* has been shown to induce COX-2 gene expression by obstructing repressive NF-κB complexes [8]. Morton et al. have shown significant up-regulation of COX-2 expression in inflamed periodontal tissues and reported possible role of inflammatory cytokines such as IL-1β and bacterial components as the underlying cause of COX-2 over-expression [10]. Based on our results, a possible explanation for up-regulation of COX-2 might be higher levels of *PACER* lncRNA. *PACER* has direct interaction with p50, which can arrange both active heterodimers with p65/RelA in the course of the normal NF-κB pathway induction, and inactive p50/p50 homodimers which are devoid of transcription activation domains [8]. NF- κB pathway has a crucial role in the pathogenesis of periodontitis [12]. Notably, most of activators of this pathway such as bacterial lipopolysaccharides, prostaglandin E2, IL-1β and TNF-α are abundantly present and active in the process of periodontitis [13]. Moreover, the *Porphyromonas gingivalis* as the most important pathogen in the periodontitis is a potent activator of NF-κB [14, 15]. Thus, modification of activation of this signaling pathway by *PACER* lncRNA might be involved in the pathogenesis of periodontitis.

Moreover, expression of *PACER* was significantly higher in female subjects compared with male subjects. Although the underlying cause of such sex-based difference in expression of this lncRNA is not clear, COX-2 as a target of this lncRNA has been shown to have sex-specific functions in control of inflammation [16]. Thus, the sex-biased up-regulation of *PACER* and the observed different levels of this lncRNA between males and females add another layer of complexity in the mechanisms of regulation of immune responses among males and females. Further molecular studies are needed to elaborate these mechanisms.

There was no significant difference in the tissue expression of *PACER* between study subgroups. Moreover, there was no significant correlation between blood and tissue levels of *PACER*. Thus, the peripheral *PACER* might exert its roles in the pathobiology of periodontitis in an independent manner from its tissue levels.

Despite the functional roles of *THRIL* in the control of TNF-α expression [7], expression of this lncRNA was not significantly different between blood/tissue samples of cases and controls. However, expression of this lncRNA was higher in blood of female subjects compared with male subjects. Although the underlying mechanism of this observation is not clear, this result is in accordance with a recently reported sex-specific role of *THRIL* in lung cancer [17].

Tissue expression of *THRIL* was correlated with blood levels of this lncRNA and with the tissue levels of *PACER*. Moreover, blood levels of these lncRNAs were correlated with each other. These data indicate the presence of similar regulatory mechanism for these lncRNAs which should be identified in future studies. Yet, expression of these lncRNAs were not correlated with age.

Taken together, we demonstrated a sex-based up-regulation of *PACER* in blood samples of patients with periodontitis which implies possible participation of this lncRNA in the pathobiology of periodontitis. However, our study has limitations regarding sample size and inaccessibility of blood samples from a number of patients and controls. Moreover, we could not assess expression of NF-κB as a putative gene interacting with these lncRNAs. Future studies should focus on identification of the interaction network between these lncRNAs and their targets in the gingival and blood tissues.

## Declarations

### Author contribution statement

S. Ghafouri-Fard and M.Taheri: Conceived and designed the experiments; Wrote the paper.

A. Sayad, B. Shams and L. Gholami: Performed the experiments; Wrote the paper.

S. Arsang-Jang: Analyzed and interpreted the data.

### Funding statement

This work was supported by a grant from 10.13039/501100005851Shahid Beheshti University of Medical Sciences.

### Competing interest statement

The authors declare no conflict of interest.

### Additional information

No additional information is available for this paper.
